# Supplementary material for: Application of CuxO-FeyOz Nanocatalysts in Ethynylation of Formaldehyde
Source: Nanomaterials (Basel). 2019 Sep 11;9(9):1301. doi: 10.3390/nano9091301 (PMC6781078; doi:10.3390/nano9091301)
Supplement: Supplementary file 1 [file nanomaterials-09-01301-s001.pdf]

Supplementary Materials

Application of Cu_xO-Fe_yO_z Nanocatalysts in Ethynylation of Formaldehyde

Haitao Li *, Lijun Ban, Zhuzhu Niu, Xin Huang, Pingfan Meng, Xudong Han, Yin Zhang, Hongxi Zhang and Yongxiang Zhao *

Engineering Research Center of Ministry of Education for Fine Chemicals, School of Chemistry and Chemical Engineering, Shanxi University, Taiyuan 030006, China

* Correspondence: htli@sxu.edu.cn (H.L.); yxzhao@sxu.edu.cn (Y.Z.); Tel.: +86-138-3457-8761 (H.L.)

1. XPS

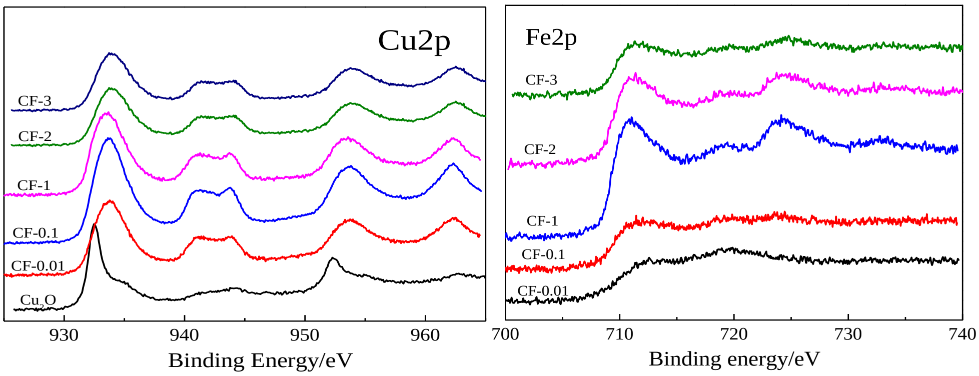

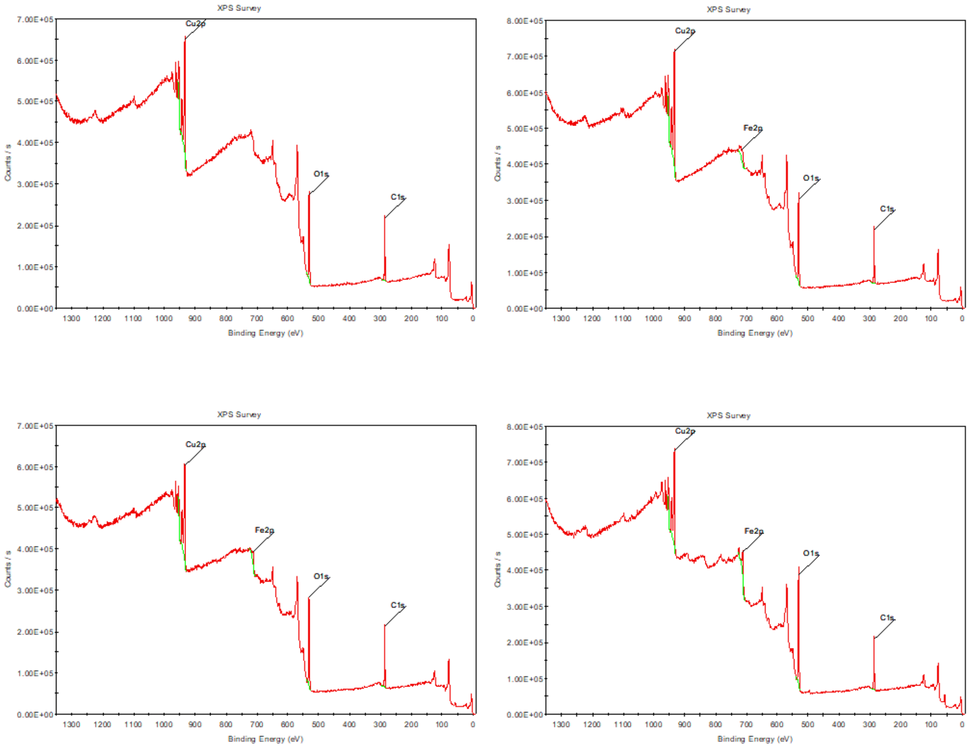

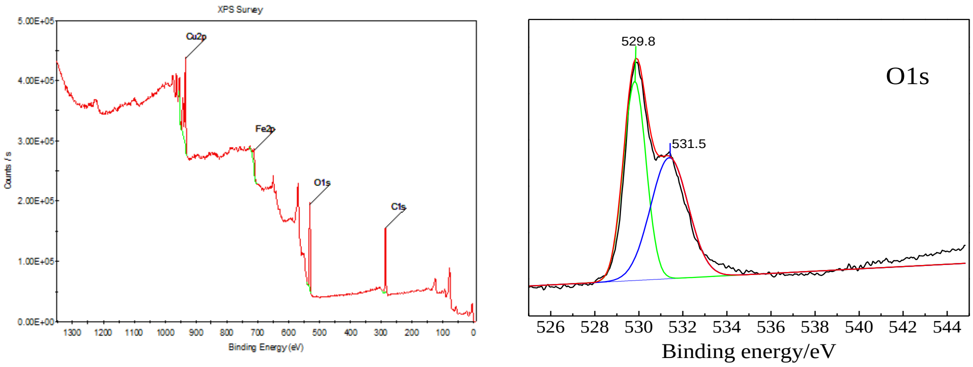

Figure S1. XPS spectra of Cu_2O and Cu_xO-Fe_yO_z with different Fe/Cu ratios.

Table S1. Surface atomic ratios in XPS spectra of Cu_2O and Cu_xO-Fe_yO_z catalysts.

Catalysts	O/Fe + Cu + O Atomic ratio
Cu_2O	29.8%
CF-0.05	38.3%
CF-0.1	43.4%
CF-0.8	46.6%
CF-2	58.7%

2. BET

Table S2. Structural and textural data for the Cu_2O and Cu_xO-Fe_yO_z catalysts.

Catalysts	BET Area(m^2/g)	Proe vlume(cm^2/g)	Proe size(nm)
Cu_2O	7.7706	0.023800	17.8306
CF-0.05	8.3051	0.021068	17.1105
CF-0.1	12.8303	0.046944	15.0914
CF-0.8	13.8774	0.04999	12.3496
CF-2	17.6027	0.052124	10.2544

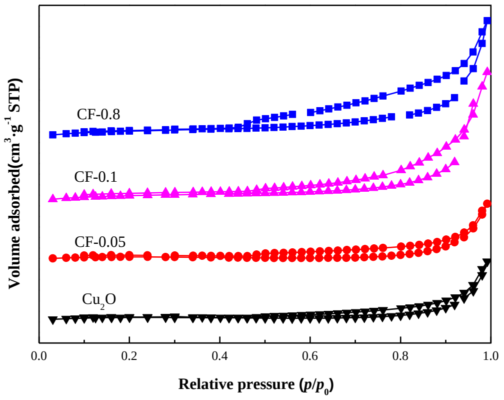

Figure S2. Structural and textural data of the Cu_2O and Cu_xO-Fe_yO_z catalysts.

3. XRD

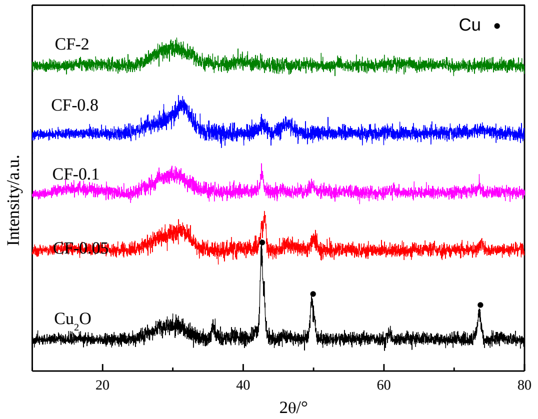

Figure S3. XRD patterns of used the Cu_2O and Cu_xO-Fe_yO_z catalysts.

4. XAES

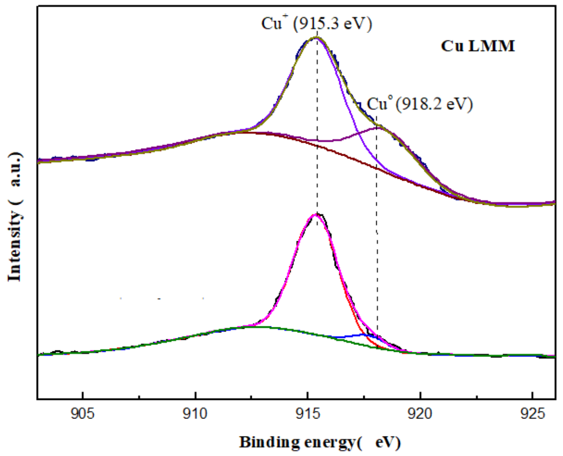

Figure S4. Cu LMM XAES spectra of the Cu_2O and Cu_xO-Fe_yO_z catalysts used after reaction.

5. Different catalyst activities

Table S3. Catalytic activity of different catalysts.

Catalysts	Initial activity (Yiled of BYD %)	6 cycles of activity (Yiled of BYD %)	References
Cu_2O	22.2%	9%	[1]
Cu_2O-TiO_2	31.3%	27%	[1]
Cu_2O-TiO_2(A/R)	28.5%/38.7%	-	[2]
CuO/N-TiO_2	56.3%	39.8%	[3]
CuO/MgO/SiO_2	59.9%	24.9%	[4]
CuO/SiO2	43.7%	22.3%	[4]

References

1. Li, H.T.; Niu, Z.Z.; Yang, G.F.; Zhang, H.X.; Wang, Z.P.; Zhao, Y.X. Support effect of Cu_2O/TiO_2 employed in formaldehyde ethynylation. *J. Chem. Ind. Eng*. **2018**, *69*, 2512–2518.
2. Wang, Z.P.; Niu, Z.Z.; Ban L.J.; Hao, Q.A.; Zhang, H.X.; Li, H.T.; Zhao Y.X. Formaldehyde ethynylation reaction over Cu_2O supported on TiO_2 with different phases. *Chemi. J. Chinese U.* **2019**, *40(2)*: 334–341.
3. Wang, Z.P.; Niu, Z.Z.; Ban L.J.; Hao, Q.A.; Zhang, H.X.; Li, H.T.; Zhao Y.X. The influence of N doping on TiO_2 supported Cu-based catalyst for formaldehyde ethynylation. *Journal of shaanxi normal university.* **2019**, *47(1)*: 99–106.
4. Wang, Z.P.; Ban L.J.; Meng, P.F.; Li, H.T.; Zhao, Y.X. Ethynylation of Formaldehyde Over CuO/SiO2 Catalysts Modified by Mg Species: Effects of the Existential States of Mg Species. Nanomaterials. **2019**, *9(8)*: 1137.
